# Case of Transplantation of Skin, at Cook Co. Hospital

**Published:** 1871-04

**Authors:** E. Powell

**Affiliations:** Attending Surgeon


Article VI.—*Case of Transplantation of Skin at Cook County Hospital.* By E. Powell, M.D., Attending Surgeon.

A. B., æt. 30, blacksmith, entered the Hospital January, 1871, for an old recurring ulcer of the leg, measuring two and a half by three inches.

Parts of its surface were deep, irregular and offensive. The patient was placed in bed, the limb elevated, and the ulcer dressed with weak solution of carbolic acid, under which it improved somewhat, but yet showed little or no disposition to heal.

It was soon determined to see what could be accomplished by the transplantation of skin upon the surface of the ulcer. Accordingly, four pieces of healthy epidermis of the size of a millet seed were removed from the inner aspect of the thigh, and inserted in a little cut made in the granulations, with the idea of giving the ulcer several points to heal from, and thereby hasten its closure; a dressing of adhesive plaster was applied. At the end of the fourth day there was nothing to be seen of transplanted skin, but on the eighth day two small isolated spots of something like cicatrization made their appearance at the points where the skin had been imbedded; this began to increase in diameter very rapidly, so much so that at the end of three weeks they were as large as a ten cent piece, appearing like islands in a sea of granulations; they were rapidly increasing in size, and two more pieces of skin were transplanted on the other portions of the ulcer. At the end of six days they were evidently commencing to send forth patches of cicatrization, which steadily progressed without interruption for nine weeks, when the ulcer was healed.

During this time there was very little effort at repair from the edges of the ulcer, the cicatrization extending almost altogether from the points of transplantation.

The only peculiarities of the cicatrix observed were its white color, and want of sensation to any foreign body.

The above brief history illustrates the points in two additional cases that were treated in the Hospital in the past six months in the same manner, with equally gratifying results, and it seems to me will work a new era in the treatment of extensive burns, by preventing the deformities which always follow such accidents.

One point is certainly plain, that the wound must heal over in a much shorter time, with much less suffering than usual, and without deformity consequent upon the contraction of the cicatrix which would otherwise follow.

